# Supplementary material for: Transfer learning for predicting acute myocardial infarction using electrocardiograms
Source: PLOS Digit Health. 2025 Oct 31;4(10):e0001058. doi: 10.1371/journal.pdig.0001058 (PMC12578225; doi:10.1371/journal.pdig.0001058)
Supplement: S1 Appendix — (PDF) [file pdig.0001058.s001.pdf]

Section A   Additional evaluation metrics

We further evaluated the effect of transfer learning for the area under the precision-recall curve (AUPR) as well as several threshold-based metrics. The thresholds were selected to maximize the Matthew correlation coefficient (MCC) on the validation set. The threshold-based metrics were MCC, accuracy, sensitivity, specificity, positive predictive value (PPV), and negative predictive value (NPV). The results for models pre-trained on age, using the full source and target datasets, are shown in Table A. The overall benefit appear similar to that of AUROC, although the magnitude of the improvement differs slightly depending on the choice of metric. In particular, the benefit of transfer learning for the RN-33M model is lower in terms of AUPR and MCC than it was for AUROC, whereas the opposite is the case for the CNN-20k model. The RN-900k model still performed the best both before and after pre-training.

The thresholded metrics (accuracy, sensitivity, specificity, PPV, and NPV) must be considered jointly, since it is trivial to maximize one at the expense of the others. Most models were improved by some metrics but degraded by others, and whether for example the 30% increase in sensitivity is worth a 16% decrease in specificity for the CNN-20k model depends on the application and circumstances of implementation. We note that all models exhibit similar tradeoffs, with the possible exception of the RN-900k model which nearly doubles the PPV with only a very marginal drop in NPV as a result of the transfer learning.

Table A. Additional metrics. Training from scratch compared to pre-training on age, using the full Source dataset. Threshold selected by maximizing MCC on the validation set. AUROC = Area under receiver operating characteristic, AUPR = Area under precision recall curve, MCC = Matthew's correlation coefficient, PPV = Positivie predictive value, NPV = Negative predictive value.

	**CNN-20k**		**RN-900k**		**RN-7M**		**RN-33M**	
	**from scratch**	**pre-trained**	**from scratch**	**pre-trained**	**from scratch**	**pre-trained**	**from scratch**	**pre-trained**
**AUROC**	0.768	0.789	0.793	0.852	0.784	0.837	0.723	0.788
**AUPR**	0.190	0.215	0.307	0.365	0.275	0.304	0.196	0.206
**Max MCC**	0.197	0.221	0.286	0.357	0.242	0.306	0.204	0.218
**Accuracy**	0.919	0.787	0.899	0.937	0.941	0.922	0.919	0.908
**Sensitivity**	0.218	0.596	0.427	0.343	0.149	0.358	0.227	0.288
**Specificity**	0.962	0.799	0.928	0.973	0.990	0.957	0.962	0.946
**PPV**	0.263	0.154	0.267	0.442	0.474	0.337	0.268	0.247
**NPV**	0.952	0.970	0.963	0.960	0.950	0.960	0.953	0.956

Section B ECG-machine metadata features

In order to investigate potential shortcuts from the ECG devices, we identified and collected the ECG location, acquisition device, acquisition software version, and cart ID as possible shortcuts. These metadata were further processed with an ordinal encoder to map them into integers and bin low frequency and missing categories into a single value. We used a minimum frequency of 4% and a maximum of 10 categories per feature. This resulted in 7 different locations (mapped from 324), 7 acquisition devices (mapped from 16), 8 software versions (mapped from 48), and two cart ID's (mapped from 354). The Pearson correlation coefficients between the metadata features and the AMI outcome is listed in Table B.

A random forest classifier was trained to predict AMI from the metadata features, and achieved an AUC of 0.509 on the validation set and 0.495 on the test set. This suggests that the metadata features are unlikely shortcuts.

Table B. Metadata feature correlations to AMI

Metadata feature	Correlation
location_-2	-0.012
location_0	-0.003
location_1	-0.001
location_2	-0.002
location_3	0.007
location_4	0.007
location_5	0.007
acquisition_device_-2	-0.012
acquisition_device_0	0.009
acquisition_device_1	0.001
acquisition_device_2	-0.002
acquisition_device_3	-0.009
acquisition_device_4	0.009
acquisition_device_5	0.001
acquisition_software_version_-2	-0.005
acquisition_software_version_0	0.010
acquisition_software_version_1	0.002
acquisition_software_version_2	0.002
acquisition_software_version_3	-0.003
acquisition_software_version_4	0.002
acquisition_software_version_5	-0.001
acquisition_software_version_6	0.000
cart_id_-2	-0.005
cart_id_0	0.005

Section C Detailed model parameters

We tried as far as possible to keep model parameters the same as reported in the original publications, but some adjustments were nevertheless required to achieve reasonable results. In particular, we have adjusted learning rates and maximum number of epochs on a case-by-case basis for each model. The batch-size was fixed to 256 for all models. The maximum number of epochs were generally between 100 and 400. The learning rate followed a one-cycle schedule which began with a linear warmup over 10 epochs, followed by a cosine decay. For the pre-training, the decay occurred over the remainder of the epochs, and for the fine-tuning, the decay lasted for 30 epochs, after which the learning rate was kept constant. The decay for the fine-tuning was chosen to coincide with the unfreezing of model parameters. Epochs, peak learning rate and final learning rate for each set of models is listed in table C. For a given model and task combination, the parameters were kept fixed for all combinations of source and target dataset sizes.

Table C. Epochs and learning rates

Model	Task	Pre-training	Max Epochs	Peak learning rate	Final learning rate
CNN-20k	Age		200	$1 \cdot 10^{-4}$	$1 \cdot 10^{-10}$
CNN-20k	Sex		200	$1 \cdot 10^{-4}$	$1 \cdot 10^{-10}$
CNN-20k	Age + Sex		200	$1 \cdot 10^{-4}$	$1 \cdot 10^{-10}$
CNN-20k	AMI		200	$1 \cdot 10^{-4}$	$1 \cdot 10^{-4}$
CNN-20k	AMI	Age	200	$1 \cdot 10^{-3}$	$1 \cdot 10^{-5}$
CNN-20k	AMI	Sex	200	$1 \cdot 10^{-3}$	$1 \cdot 10^{-5}$
CNN-20k	AMI	Age + Sex	200	$1 \cdot 10^{-3}$	$1 \cdot 10^{-5}$
RN-900k	Age		100	$1 \cdot 10^{-3}$	$1 \cdot 10^{-5}$
RN-900k	Sex		100	$1 \cdot 10^{-3}$	$1 \cdot 10^{-5}$
RN-900k	Age + Sex		100	$1 \cdot 10^{-3}$	$1 \cdot 10^{-5}$
RN-900k	AMI		100	$1 \cdot 10^{-3}$	$1 \cdot 10^{-5}$
RN-900k	AMI	Age	400	$1 \cdot 10^{-3}$	$1 \cdot 10^{-5}$
RN-900k	AMI	Sex	400	$1 \cdot 10^{-3}$	$1 \cdot 10^{-5}$
RN-900k	AMI	Age + Sex	400	$1 \cdot 10^{-3}$	$1 \cdot 10^{-5}$
RN-7M	Age		100	$5 \cdot 10^{-4}$	$5 \cdot 10^{-10}$
RN-7M	Sex		100	$5 \cdot 10^{-4}$	$5 \cdot 10^{-10}$
RN-7M	Age + Sex		100	$5 \cdot 10^{-4}$	$5 \cdot 10^{-10}$
RN-7M	AMI		100	$5 \cdot 10^{-4}$	$5 \cdot 10^{-6}$
RN-7M	AMI	Age	400	$1 \cdot 10^{-3}$	$1 \cdot 10^{-5}$
RN-7M	AMI	Sex	400	$1 \cdot 10^{-3}$	$1 \cdot 10^{-5}$
RN-7M	AMI	Age + Sex	400	$1 \cdot 10^{-3}$	$1 \cdot 10^{-5}$
RN-33M	Age		100	$5 \cdot 10^{-4}$	$5 \cdot 10^{-10}$
RN-33M	Sex		100	$5 \cdot 10^{-4}$	$5 \cdot 10^{-10}$
RN-33M	Age + Sex		100	$5 \cdot 10^{-4}$	$5 \cdot 10^{-10}$
RN-33M	AMI		200	$5 \cdot 10^{-4}$	$5 \cdot 10^{-6}$
RN-33M	AMI	Age	200	$1 \cdot 10^{-3}$	$1 \cdot 10^{-5}$
RN-33M	AMI	Sex	100	$1 \cdot 10^{-4}$	$1 \cdot 10^{-6}$
RN-33M	AMI	Age + Sex	200	$1 \cdot 10^{-3}$	$1 \cdot 10^{-5}$

Section D Model architectures

The following sections illustrate the architectures and the most important parameters of each base model.

CNN-20k

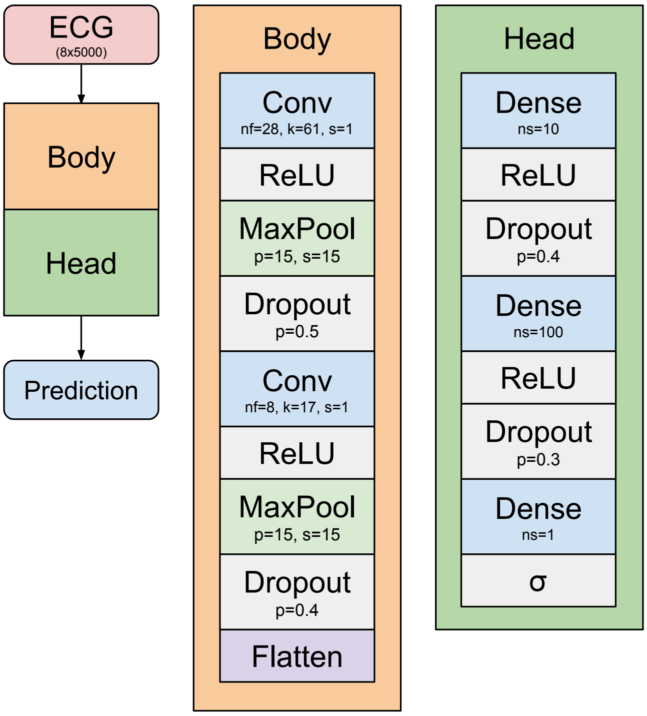

Fig A. Schematic overview of the CNN-20k model from [1]. nf=number of filters, k=kernel size, p=pool size in pooling layer and probability of dropout in dropout layer, s=stride, ns=number of output samples, Dense=Fully connected layer, Conv=1D Convolutional layer, ReLU=Rectified Linear Unit, σ=logistic function.

RN-900k

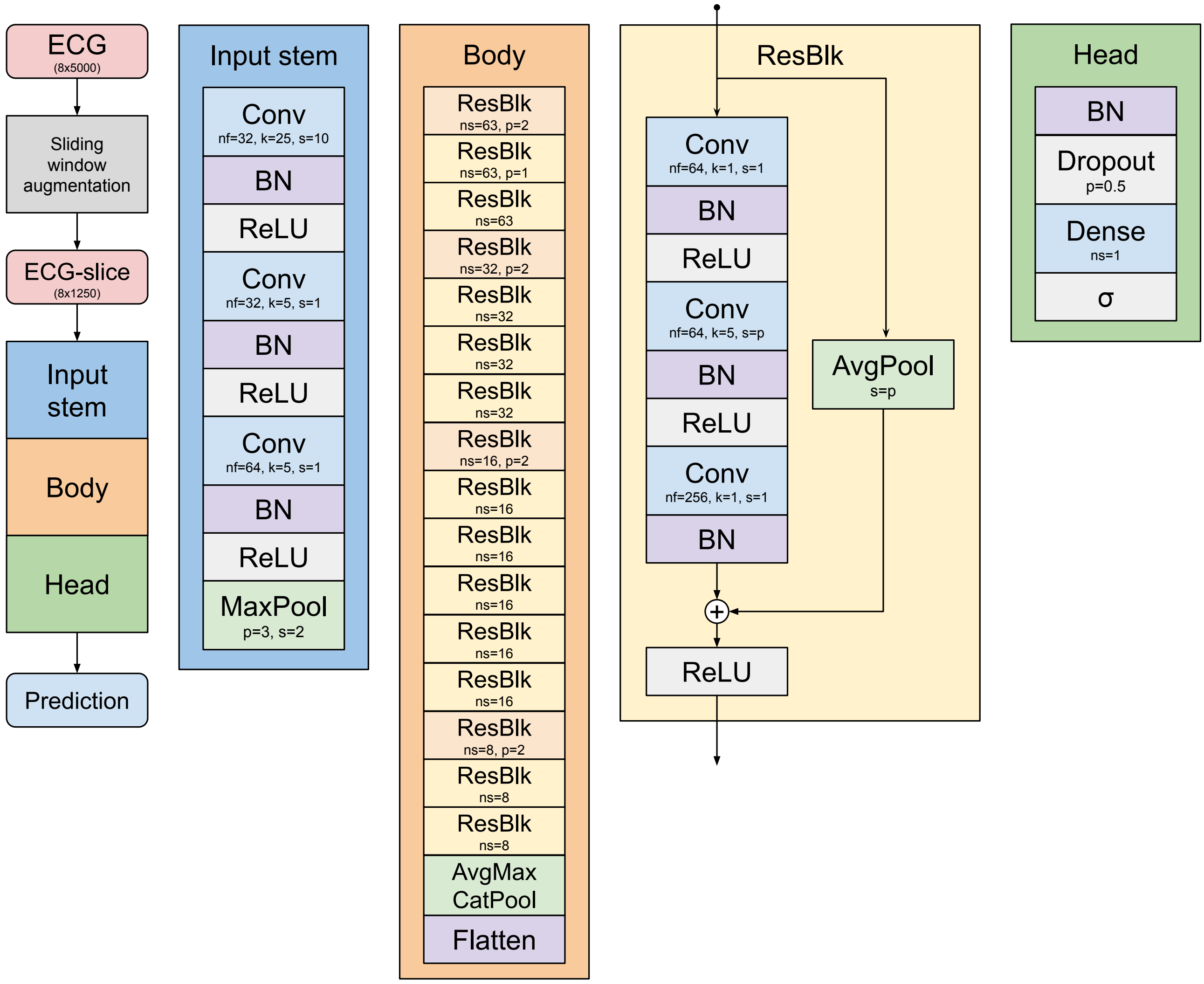

Fig B. Schematic overview of the RN-900k model from [2]. During training, the sliding window augmentation randomly crops each input batch of ECGs. During validation and testing, the input ECG is sliced ten times, and the resulting predictions are averaged. The residual blocks (ResBlk) with a pool size of 2 (indicated by slightly darker yellow/orange) represent a downsampling of the input by a factor 2. The AvgMaxCatPool is a layer that concatenates the results of an average and max-pool layer. nf=number of filters, k=kernel size, p=pool size in pooling layer and probability of dropout in dropout layer, s=stride, ns=number of output samples, BN=Batch Normalization, Dense=Fully connected layer, Conv=1D Convolutional layer, ReLU=Rectified Linear Unit, σ=logistic function.

RN-7M

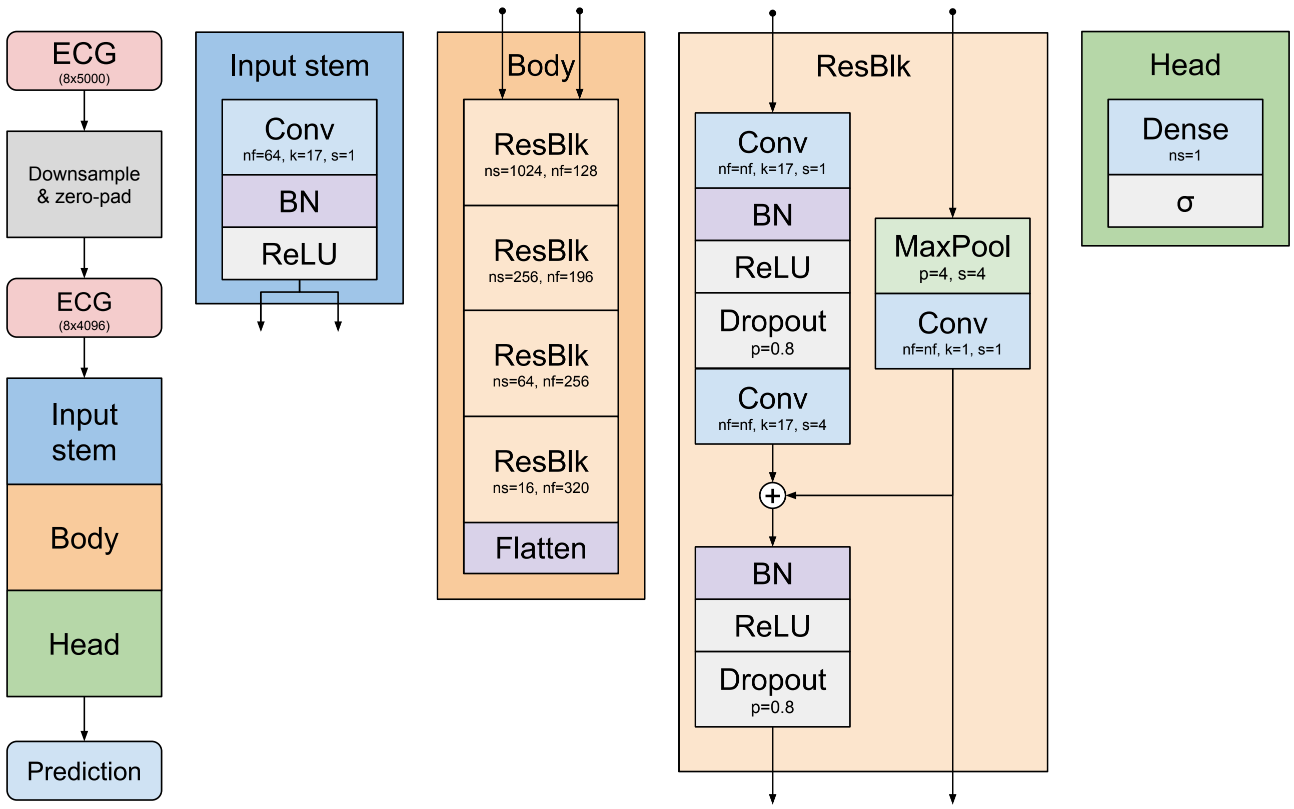

Fig C. Schematic overview of the RN-7M model from [3]. The downsampling step downsamples the input signal from 500 Hz to 400 Hz. nf=number of filters, k=kernel size, p=pool size in pooling layer and probability of dropout in dropout layer, s=stride, ns=number of output samples, BN=Batch Normalization, Dense=Fully connected layer, Conv=1D Convolutional layer, ReLU=Rectified Linear Unit, σ=logistic function.

RN-33M

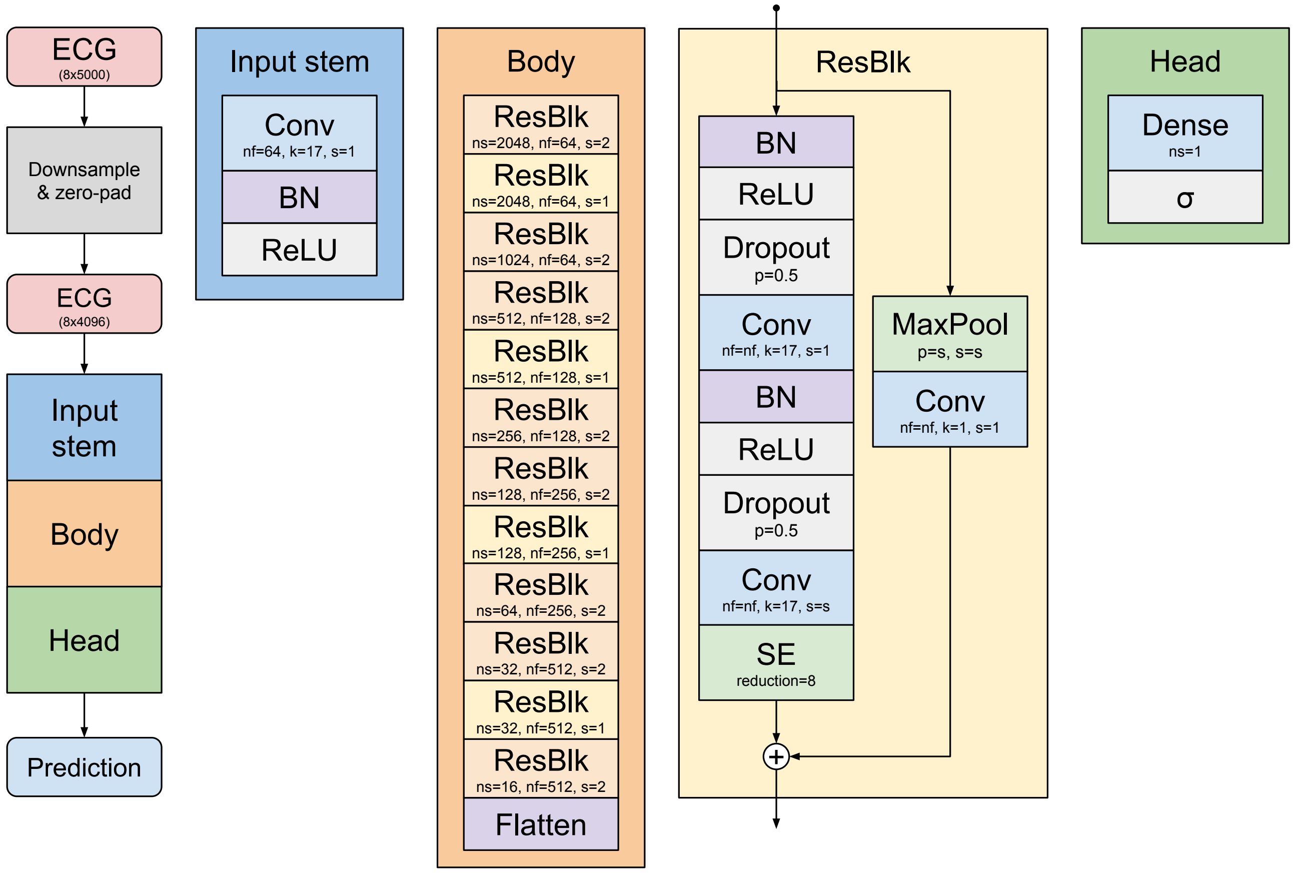

Fig D. Schematic overview of the RN-33M model from [4]. The downsampling step downsamples the input signal from 500 Hz to 400 Hz. The residual blocks (ResBlk) with a stride of 2 (indicated by slightly darker yellow/orange) represent a downsampling of the input by a factor 2. nf=number of filters, k=kernel size, p=pool size in pooling layer and probability of dropout in dropout layer, s=stride, ns=number of output samples, BN=Batch Normalization, Dense=Fully connected layer, Conv=1D Convolutional layer, ReLU=Rectified Linear Unit, σ=logistic function.

Section E   Model training times

All models were trained on machines using Nvidia GeForce 3090 GPUs, with enough RAM to load the entire ECG dataset at once (no streaming from disk was necessary). The code was written in Python 3.11, using Pytorch 2.0 and Tensorflow 2.14 libraries. Table D shows the training times for pre-training on age, using the full source dataset. Perhaps somewhat counter-intuitively, the training times are not simple linear functions of model parameters, which may be explained by a number of factors. Importantly, the model architectures, though somewhat similar, have substantial differences. In particular, the RN-900k network begins with a sliding-window augmentation that effectively only uses 25% of the input signal at a time, and follows with a convolutional layer with a stride of 10, further reducing the size of the input signal, which helps to speed up training. The benefit of GPU acceleration also strongly depends on model architecture, in our case disproportionately helping the larger models.

Finally, we point out that we have not optimized any of the models for speed; it is plausible that substantial speed-ups could be achieved by tweaking parameters and model architectures without meaningfully affecting classification performance, though we have not explored this topic in the present study.

Table D. Model training times

Model	Conv. layers	Parameters	Epochs	Total training time (h)	Training time (ms/ECG)
CNN-20k	2	20 479	200	20.4	0.46
RN-900k	51	892 449	100	3.1	0.14
RN-7M	9	6 784 561	100	18.4	0.84
RN-33M	25	33 062 569	100	23.6	1.07

Overview of model training times for predicting age on the full source dataset (n =794 694). Times are elapsed real time, including memory management and validation set inference after each epoch.

References

1. Nyström A, Olsson de Capretz P, Björkelund A, Lundager Forberg J, Ohlsson M, Björk J, et al. Prior electrocardiograms not useful for machine learning predictions of major adverse cardiac events in emergency department chest pain patients. Journal of Electrocardiology. 2024;82:42–51. doi:10.1016/j.jelectrocard.2023.11.002.

2. Mehari T, Strodthoff N. Self-supervised representation learning from 12-lead ECG data. Computers in Biology and Medicine. 2022;141:105–114. doi:10.1016/j.compbiomed.2021.105114.

3. Ribeiro AH, Ribeiro MH, Paixão GMM, Oliveira DM, Gomes PR, Canazart JA, et al. Automatic Diagnosis of the 12-Lead ECG Using a Deep Neural Network. Nature Communications. 2020;11(1):1760. doi:10.1038/s41467-020-15432-4.

4. Gustafsson S, Gedon D, Lampa E, Ribeiro AH, Holzmann MJ, Schön TB, et al. Development and validation of deep learning ECG-based prediction of myocardial infarction in emergency department patients. Scientific Reports. 2022;12(1):19615. doi:10.1038/s41598-022-24254-x.
