# Supplementary material for: Transfer learning for predicting acute myocardial infarction using electrocardiograms
Source: PLOS Digit Health. 2025 Oct 31;4(10):e0001058. doi: 10.1371/journal.pdig.0001058 (PMC12578225; doi:10.1371/journal.pdig.0001058)
Supplement: Attachment — Submitted filename: Response to Reviewers.pdf [file pdig.0001058.s002.pdf]

Response to Reviewers

Axel Nyström

June 2025

1 Reviewer 1

1.1

I suggest the authors include more information on the 'Usability of the model in the context of current care' (item 27 in the TRIPOD+AI statement). Even if the implementation is beyond the scope of this paper, the ultimate goal of a clinical prediction model should be to do so, so discussion of attention points would be relevant in my opinion.

We agree, and have added a new subsection called "Usability in the context of current care".

1.2

From a clinical perspective, when prediction models are used on an individual basis often with some threshold values triggering actions (e.g. if risk is over 10%, then ...), calibration of the model describing agreement between predicted risk and observed events is crucial. (This aspect is way underreported in the machine learning literature.) I suggest the authors report calibration plots and metrics like calibration-in-the-large and calibration slope (see e.g. CalibrationCurves R package, not sure about corresponding tools in Python).

We agree that model calibration is an important aspect of clinical prediction models. In the present study however, we are mainly interested in how model performance is affected by transfer learning, which is orthogonal to issues of calibration. In future studies, we aim to use the results presented here to develop better models, and as we approach a point where models may be tested in a clinical setting, it would absolutely be warranted to consider aspects of calibration and how they apply to the specific clinical circumstances. We have added a paragraph about this in the limitations section.

1.3

It would be clinically relevant to investigate the results from an algorithmic fairness perspective (TRIPOD+AI items 3c, 14, 23a). Given the well-known sex difference in cardiovascular risk, also mentioned by the authors, that would be an obvious sensitive attribute or subgrouping to report the results by and investigate the fairness of the model. Would there be other relevant factors (e.g. socio-economic status, ethnicity, if there is data on these)?

This is an excellent suggestion, thank you! We examined the transfer-learning effects when stratifying on sex and age quartiles, for the models trained using the full source and target data sets, and have added relevant sections in the manuscript (Fairness in the Methods chapter and Subgroup analysis in the Results and discussion chapter).

1.4

Reporting model performance by sex (just the main results, not for different %s of source and target data) could also shed light on another aspect that has raised concerns around deep learning models recently i.e. shortcut learning. Given the good discrimination for sex, and higher cardiovascular risk among male patients, I wonder how much are the results explained by the model predicting sex as a shortcut and not the actual task i.e. AMI. It would be relevant to see the distribution of the outcome not only by splits, but also by sex within the splits and if the authors think that there could be any other similar factors. These tables could be part of the supplementary materials. Please consider what other shortcuts would be feasible in the dataset and discuss their potential impact on the results. E.g. Are different ECG devices used in different hospitals where the risk of AMI is lower/higher? Can the type of the machine be detected?

Thank you for pointing out the importance of assessing shortcut learning, this is something we hadn't considered for this paper. We looked into potential shortcuts from ECG-machine related metadata, as well as unwanted shortcuts from age and sex. We have added corresponding sections in the Materials and methods chapter (section "Shortcut learning"), and corresponding results in the Results chapter ("Shortcut learning results").

We also included (in the new "Subgroup analysis" section) a breakdown of the outcome distribution by sex and age quartiles within each split.

1.5

From an epidemiological perspective, I am curious what happened to the people in the analysis who died within 30 days? How many were

there and how were they handled in the analysis if the cause of death was / was not AMI?

It's a good question, and it's always interesting to consider various corner cases and potential mismatches between different data sources. In this paper we opted to simplify and not consider deaths in our analysis or definition of the target label. In other words, only the diagnosis of AMI in the national in-patient register was used, regardless of whether the patient ended up dying within 30 days, and regardless of cause of death. A combined outcome of AMI or death might arguably have been more suitable, although it would come at the cost of additional complexity which we felt was not warranted for this study.

Looking at the numbers for the target dataset (44370 visits), we found 549 deaths within 30 days. Of these, 146 were marked with AMI as primary cause of death. And of those, 44 were not reported as AMI to the national patient register, so it's possible that some of them should be considered false negatives with respect to our AMI label. All things considered, though adjusting the labels might alter the model performances, we don't believe this would have a meaningful impact on the overall conclusions of the paper, or the effect of transfer learning.

1.6

Also, were people/visits with missing data different from those without? What can be the main reasons for missingness and could this affect the generalizability of the results?

The only exclusion criteria for the target dataset was the lack of an index ECG of sufficient quality, in this case basically only meaning that there were no missing ECG leads. This was poorly described in the manuscript, and has been revised. The SEM database in turn includes everybody who arrived at any ED in the entire region, excluding only patients without Swedish personal number, patients under 18, patients who left against medical advice, and patients who explicitly asked to be removed from the dataset. We believe this is about as close as it gets to a representative view of patients with chest pain at the emergency department.

Speaking of generalizability, we have also (in response to Reviewer 3, comment 5), performed an external validation on the PTBXL database, the result of which suggests that our transfer learning approach can generalize across different databases.

2 Reviewer 2

2.1

Transfer learning has a broad scope. However, only the fine-tuning strategy is studied and described in Lines 221-231. Other transfer learning strategies, such as knowledge distillation, should be discussed or even evaluated in experiments.

It is absolutely true that transfer learning is a broad topic that has been well-studied in many applications. Many variations exist, and the fine-tuning strategy is perhaps one of the simplest of the popular versions. We also agree that evaluating other strategies would be interesting, but that doing so would needlessly broaden the scope of the current paper, and is thus more suitable as a follow-up study. We have added a paragraph in the Limitations section to clarify our position.

2.2

Additional performance metrics (e.g., precision, recall) should be reported in experiments besides the currently reported AUC and accuracy.

Thank you for this suggestion. We have added a section in the supplement that evaluates the main results (100% source and 100% target data, comparing pre-training on age with no pre-training) with additional performance metrics. Specifically, we evaluate the area under the precision-recall curve, and then select the threshold that maximizes the Matthew correlation coefficient (MCC) on the validation set. We then use that threshold to evaluate the following metrics (on the test set): accuracy, MCC, sensitivity (recall on the positive class), specificity (recall on the negative class), positive predictive value (precision on the positive class), and negative predictive value (precision on the negative class).

2.3

As acknowledged in the current paper (Line 319), the choice of hyperparameters is not studied. Is there any special reason for this?

The main reason for not tuning hyper-parameters was the computational cost: we argue (see lines 327-330) that for the comparisons to be "fair", we would have to re-tune each model for each task on each dataset size, which would incur an unreasonable computational cost for what we expect to be a relatively minor gain.

3 Reviewer 3

3.1

Outdated Transfer Learning Approach. Relies solely on supervised pretraining for sex and age classification instead of modern self-supervised learning (SSL) or contrastive learning approaches. No comparison with ECG foundation models such as: PTB-XL pre-trained models, Self-supervised ECG models (Mehari et al. 2022, 2023), Contrastive Predictive Coding (CPC) for ECGs, ECG-BERT models, Time-series Transformers for ECG (Hyena, Perceiver-IO, etc.)

We appreciate the suggestion and understand the interest in exploring more advanced forms of transfer learning in this context. However, we believe that such analysis falls out of scope of the current study, which quantifies the usefulness of a single, comparatively simple transfer learning approach for predicting AMI with ECGs. The broader literature on transfer learning is quite extensive, and doing justice to a comparison between many of these approaches would introduce substantial additional complexity. We agree that this would be a valuable direction for future research, and have added a paragraph in the Limitations section relating this view.

3.2

Weak Pretraining Task Choice (Age and Sex). Age and sex classification does not provide meaningful ECG features for AMI prediction. Self-supervised learning captures physiological representations better than simple demographic features.

We somewhat disagree that age and sex are not meaningful ECG features for AMI prediction: in fact, they are quite important for the evaluation of AMI in clinical practice, and typically help to improve ML-based predictions. It could be argued that other pre-training tasks might work better, but in our view, a more thorough investigation of this falls out of scope for the current paper. We don't claim that age and sex are optimal choices, but although they are convenient for several reasons (they are universally available, highly accurate labels representing non-trivial tasks with somewhat strong correlation to the target task), the main takeaway here is that it works. Although other approaches might indeed yield even better results, the same is true for many modeling choices, and to establish optimality is inherently challenging. Our goal here is not to claim that this is the best possible approach, but to demonstrate that it is effective, practical, simple, and based on clinically meaningful features.

3.3

Lack of Clinical Interpretability and Explainability. No analysis on feature importance or interpretability (e.g., DeepSHAP, Grad-CAM, attention maps). Could benefit from: Saliency map visualizations, SHAP value analysis, Comparison with physician interpretations.

We appreciate the comment and fully agree that clinical interpretability and explainability are important and relevant topics – particularly in the context of machine learning in health care. These concepts are central to trust, usability, and clinical adoption. That said, we believe that interpreteability does not exist in a vacuum – it depends heavily on context. For whom should the model be explainable, and in what way? This is a non-trivial question. The needs of researchers, developers, clinicians, and patients can differ greatly, and the effectiveness of commonly used tools such as saliency maps or SHAP values in meeting these needs remains an open question.

We do agree that exploring how interpretability is influenced by transfer learning would be interesting and potentially valuable, and we consider this a promising direction for future work. Among the reviewer's suggestions, we particularly see the value in comparing model outputs with physician interpretations. Unfortunately, the SEM database does not include clinical interpretations of the ECGs, which limits our ability to perform such a comparison.

Given these limitations and the current focus of the study, we believe that a detailed exploration of interpretability and explainability lies outside the scope of this paper. However, we will consider addressing this in future work.

3.4

Lack of Robustness Tests. No evaluation of generalization across demographics, hospitals, or ECG devices. Does not assess performance under missing leads or noisy ECGs.

Although we did consider certain aspects of robustness by utilizing both a random and chronological test split, this analysis was unfortunately sidelined to make room for the main comparisons. We agree that robustness is a very important topic, and that more would have to be done before implementing a model like this in a real clinical setting. We have added a section (Usability in the context of current care), to address this point.

Additionally we have introduced a section examining the results stratified on age and sex, as well as a new section on the topic of shortcut learning, in which we found that ECG location, device, and software version were uncorrelated with the AMI outcome.

We are less concerned about very noisy ECGs, for the simple reason that if the ECG is too noisy, a clinician would simply take a new ECG.

3.5

Lack of External Validation. Entirely based on Skåne Emergency Medicine (SEM) database. No validation on independent datasets (e.g., PTB-XL, MIMIC-III, CODE-15).

Thank you for this excellent feedback, it has prompted us to further evaluate our methods on different datasets. We have done so on PTBXL, and found that our approach generalizes well, which we believe substantially strengthens our conclusions and the paper as a whole. Specifically, we have trained our four models to predict AMI in PTBXL, with and without pre-training on age in SEM. We also pre-train the RN-900k network on age in PTBXL, and fine-tune on SEM. We have added an External validation section describing our results.

3.6

Poor Comparison to Non-Transfer Learning Baselines. Only compares to models trained without pretraining. Should compare to: End-to-end models trained directly on AMI data, Models pre-trained using self-supervised or contrastive learning methods.

We appreciate the thoughtful comment and fully agree that comparisons to other transfer learning approaches, including self-supervised learning and contrastive learning, are highly relevant and of great interest to the field. These methods represent promising directions for improving model performance and warrant careful exploration.

However, we chose to focus this work on evaluating the utility of transfer learning from a simple, clinically meaningful prediction task. Expanding the scope to include a full comparison between other transfer learning strategies would significantly broaden the study and require additional experimentation beyond what is feasible within the current paper.

That said, we acknowledge the importance of this comparison and have added a paragraph in the discussion section to clarify our reasoning and suggest this as a valuable direction for future work.
