# Supplementary material for: Transfer learning for predicting acute myocardial infarction using electrocardiograms
Source: PLOS Digit Health. 2025 Oct 31;4(10):e0001058. doi: 10.1371/journal.pdig.0001058 (PMC12578225; doi:10.1371/journal.pdig.0001058)
Supplement: Attachment — Submitted filename: Response_to_Reviewers_auresp_2.pdf [file pdig.0001058.s003.pdf]

Response to Reviewers

Axel Nyström

September 2025

1 Reviewer 1

1.1

Although the presented analysis and results are interesting (and reassuring for metadata), I am not sure why the authors took a different approach for age and sex. To be honest, I am not 100% sure what the latter approach is, so this could be explained more both in the Methods and the Table 7 legend (as the table should be self-explanatory). Are age and sex combined with the predicted probabilities as features? What I had in mind was much simpler and similar to what they did with the metadata i.e. to fit a logistic regression with age and sex as the sole predictors to create a benchmark performance and see whether deep learning, with or without transfer learning, beats that as expected.

It is true that the ECG models, even without pre-training, are better than age and sex (which achieves an AUC of 0.712). But since there is predictive power in age and sex, we became concerned that the benefit of our transfer learning strategy was only a result of the models learning to estimate age and sex, as opposed to becoming more competent at ECG analysis in a more general sense. If this was the case, we could just add age and sex as features rather than doing the much more time-consuming pre-training. We therefore felt that we needed to do some additional testing. We have re-worded the second paragraph of the shortcut learning section in an attempt to clarify this. The paragraph now reads:

"Another potential shortcut is in the pre-training task itself, i.e. age and sex. Although both age and sex are useful predictors for AMI on their own, their usefulness for transfer learning would be diminished by the extent to which we could get similar improvements by simply adding age and sex as input features directly. In other words, it could be that the observed improvement from our transfer learning strategy is merely a result of the models being able to infer useful features (age and sex), rather than a more general ability to interpret ECGs.

This would constitute an unwanted case of shortcut learning, because the gains from transfer learning would be nullified by the introduction of simple features that were easily available all along. We test this potential shortcut by fitting logistic regression models to age and sex together with the *predictions* from the ECG models, either with or without transfer learning. If logistic regression performs as good with the pre-trained model predictions as with predictions from models trained from scratch, this would indicate that age and sex were shortcuts, greatly diminishing the usefulness of our transfer learning approach."

We have also improved the title and legend of Table 7.

2 Reviewer 2

The revision is fine; the authors have acknowledged the limitations of the study, such as the lack of evaluation of modern transfer learning strategies.

3 Reviewer 3

3.1

It is crucial to confirm that there is no patient overlap between the pre-training (source) and fine-tuning (target) sets. While the authors mention that patients in the source dataset were excluded from the target, stronger emphasis on this separation is warranted, especially due to the known confounding role of age and sex in AMI risk. This is necessary to eliminate potential data leakage and ensure fair generalization.

We agree this is an important detail. We added the following sentence (on line 137), to emphasize this: "There is, in other words, no patient overlap between the source and target datasets."

3.2

The choice of AUC as the primary evaluation metric for AMI prediction, despite the class imbalance (only 6% AMI prevalence), is suboptimal. Precision-recall AUC would better reflect performance in this imbalanced setting. Consider adding precision-recall curves or PR-AUC as a complementary metric.

We have already included precision-recall AUC among the additional metrics listed in Table 1 of the supplement. We would like to note, however, that the view that PR-AUC is more suitable than ROC-AUC for unbalanced data has been challenged, for example by Richardson et al. [1]

3.3

The manuscript would benefit from a clearer comparative discussion of how the proposed supervised pre-training (age/sex prediction) fares against unsupervised (e.g., autoencoder by Jang et al [16], self-supervised or contrastive pre-training methods (e.g., [21]). Could the authors quantify or qualitatively discuss relative benefits or limitations of supervised vs. un/self-supervised strategies in this context?

We agree that such analysis would be interesting, however we think it falls out of scope for this paper. This view is reflected in the final paragraph of the limitations section. See also review comments 2.1, 3.1, and 3.6 from the initial review round, and our responses there.

3.4

The performance comparison across different models may be confounded by differing levels of architectural optimization and pre-training compatibility. This difference should be highlighted as a limitation and could be addressed in future work via more harmonized model baselines.

We agree that this is a substantial limitation in the present work, which we have addressed in the second paragraph of the limitations section. We agree it would be a good idea to attempt to harmonize the model baselines, and have added the following sentence to the end of the second paragraph (line 455) in the limitations section to reflect this:

"Future research would ideally be able to utilize more harmonized model baselines in order to better elucidate the effects of transfer learning."

3.5

The external validation on PTB-XL is appreciated, but the results could be further elaborated. For example, how consistent is the model performance across label distributions in PTB-XL vs SEM?

We thank the reviewer for this suggestion. The PTB-XL dataset contains 71 different outcomes, of which many are not available in the SEM dataset, thus making a direct comparison unfeasible. Conversely, we believe that a more in-depth analysis of transferring from SEM to PTB-XL with regards to other outcomes would stray too far from the main objective of our paper. We think that maintaining a focus on AMI will best serve the clarity and purpose of the study.

3.6

Subgroup analysis reveals interesting trends. These insights should be explored further, especially considering the low AMI incidence in these groups. What might be the implications for clinical deployment in underrepresented populations?

We thank the reviewer for this comment. In our subgroup analysis, we found that the benefit of transfer learning disproportionately affected younger patients and women, who both have a lower incidence of AMI and are often underrepresented. This suggests that pretraining may help improve fairness of the model by strengthening performance where it is usually weaker. We agree that these findings highlight important considerations for clinical deployment, as improved performance in such subgroups could support more equitable deployment of the model in practice. We have extended our discussion of the subgroup analysis to highlight these reflections:

"Overall, our findings indicate that transfer learning might be useful for improving model fairness in groups with lower incidence. This may represent a fruitful avenue for further research into equitable deployment of clinical decision support tools based on deep learning. "

3.7

The shortcut learning analysis is an interesting component. However, I do not see the conclusion of "the predictions from the pre-trained models improved the logistic regression model" is made. In Table 7, you have "No pre-training", "Age", "Sex", and "Age & "Sex". Are the latter 3 the resulting accuracy of logistic regression? Since you have the 4 deep learning models as 4 rows, don't you need to have logistic regression as the fifth row for comparison?

Thank you for highlighting the need to clarify the title and structure of Table 7. The table was meant to explore whether our observed improvements from transfer learning was an effect of age and sex being used as shortcuts to the AMI outcome. We have revised the title to be more explanatory and clarified the meaning of the columns. The description of the method has also been clarified in the manuscript, following comments from reviewer 1 (comment 1). We believe these changes have improved the clarity of the table and adequately address the reviewer's concern.

3.8

Minor grammar/wording issues in places (e.g., 'the patients in the source dataset is...' should be 'are')

Thank you. We have updated the manuscript and ran additional grammar

and spell checks.

3.9

In the exclusion diagram, it would be helpful to clarify the logic in plain language.

We agree, and have added a plain text description to the figure caption, which now reads:

”Visualization of exclusion criteria. SEM refers to the Skåne Emergency Medicine cohort, which includes all ED visits between 2017-01-01 and 2018-12-31 from 8 hospitals in Skåne, Sweden [2]. The target dataset is based on chest-pain visits, and divided into training, validation and test sets in a patient-wise manner. The test set is divided into a random and a temporal set, with the temporal set including all visits after a cutoff date (2018-10-05). Visits prior to the cutoff were excluded for patients in the temporal test set. The source dataset includes all ECGs from patients that were not included in the target dataset. CP = chest pain, ED = emergency department.”

3.10

Consider including model training time or FLOPs in Table 2 to contextualize complexity-performance trade-off.

Thank you for this idea. We have now included training times, but decided to add them to the supplement (together with a discussion), so as not to distract too much from the main manuscript. See Appendix, section 5.

3.11

Several useful details are in the S1 Appendix. Reference them more clearly in the main text.

We have updated the references to be more specific.

3.12

What’s the self-supervised learning objective in Mehari et al [22] S4 pre-training regime?

Mehari *et al.* uses a contrastive predictive framework in which the latent space representation of a slice of the ECG is used to predict the latent space representation of a future slice of the same ECG. The loss function used is called InfoNCE, described by van den Oord *et al.* [3]

References

1. Richardson E, Trevizani R, Greenbaum JA, Carter H, Nielsen M, Peters B. The receiver operating characteristic curve accurately assesses imbalanced datasets. Patterns. 2024;5(6):100994. doi:10.1016/j.patter.2024.100994.

2. Ekelund U, Ohlsson B, Melander O, Björk J, Ohlsson M, Lundager Forberg J, et al. The Skåne Emergency Medicine (SEM) cohort. Scandinavian Journal of Trauma, Resuscitation and Emergency Medicine. 2024;32(1):37. doi:10.1186/s13049-024-01206-0.

3. van den Oord A, Li Y, Vinyals O. Representation Learning with Contrastive Predictive Coding; 2019.
